# Living on Oral Anticoagulants: Duke Anticoagulation Satisfaction Scale Results

**DOI:** 10.3390/jcm12247574

**Published:** 2023-12-08

**Authors:** Antonella Mameli, Alessandro Sestu, Francesco Marongiu, Doris Barcellona

**Affiliations:** 1 Department of Internal Medicine, Azienda Ospedaliero-Universitaria di Cagliari, 09123 Cagliari, Italy; a.mameli@aoucagliari.it (A.M.); a.sestu@aoucagliari.it (A.S.); 2 Department of Medical Science and Public Health, University of Cagliari, 09123 Cagliari, Italy; francesco.marongiu@unica.it

**Keywords:** vitamin K-antagonists, direct oral anticoagulants, quality of life, DASS instrument, thrombosis and hemostasis unit

## Abstract

Background: Direct oral anticoagulants (DOACs) are widely used in patients with atrial fibrillation and venous thromboembolism. The lack of the need for laboratory monitoring and a better safety than vitamin K antagonists (VKAs) has probably changed the quality of life of patients on these oral anticoagulants. This was a real-life prospective observational cohort study. The aim was to evaluate if a long-term treatment with DOACs could offer a better quality of life than VKAs. Moreover, age, gender, education level, time in therapeutic range for VKAs, taking medication once or twice a day for DOACs, the total daily number of medications and thrombotic and bleeding complications were considered as variables probably associated with the quality of life of these patients. Methods: Between January and December 2021, the Duke Anticoagulation Satisfaction Scale (DASS) 25-items was administered as an interview to patients on either VKAs or DOACs therapy. During the follow-up period, all of the patients were closely monitored to evaluate possible bleeding and thrombotic events. Results: The analysis included 300 outpatients treated with VKAs and 254 treated with DOACs. In general, the quality of life was better in patients taking DOACs (DASS total score: DOACs = 44.7, 42.9–46.5 vs. VKAs = 51, 49.2–52.8, *p* < 0.0001) as the daily-life limitations, hassles and burdens and the psychological impact were less important than in patients on VKAs therapy. Conclusions: VKAs negatively influence the daily-life of the patients in terms of both less satisfaction and time-consuming tasks. DOACs confer a better quality of life even if some concerns emerge from not knowing how their therapy is working.

## 1. Introduction

Oral anticoagulant therapy is used worldwide in the prevention and treatment of venous and arterial thrombosis. In Italy, vitamin K antagonists (VKAs), were the only drugs prescribed up to 2013 when direct oral anticoagulants (DOACs) became available for the pharmacological market. The most important differences between these two classes of drugs are that DOACs do not require a coagulative test monitoring and the quality of the therapy is independent by the doctors’ ability to handle the weekly dosage but closely dependent by patients’ adherence and persistence. Moreover, both clinical trials and real-life studies have shown that DOACs safety is greater than that of coumarin derivatives [1,2,3,4,5,6,7,8,9]. These differences have allowed DOACs to be the most widely used drugs today in patients affected by atrial fibrillation and venous thromboembolism, and their indications are growing over the years [10].

However, VKAs still retain an indication in complex patients at high thrombotic risk as those with moderate-severe mitral stenosis [10,11], antiphospholipid syndrome [12], severe renal failure with a glomerular filtration rate <30 mL/min for direct thrombin inhibitor and <15 mL/min for anti-Xa inhibitors [10], cirrhotic patients with a Child-Pugh score of B or C [10], mechanical prosthetic heart valves [13].

The majority of oral anticoagulated patients take these medications throughout their life, so adherence to the therapy and quality of life (QoL) are two important aspects to consider since they can affect the efficacy and safety of the treatment [14,15].

It has been shown that, in general, chronic therapies can cause a lowering of the QoL and this in turn constitutes a risk of poor adherence over time [16]. The possibility of using DOACs in the prevention and treatment of thrombosis in the two most frequent pathologies such as atrial fibrillation and venous thromboembolism has changed the clinical scenario and probably the QoL of these patients.

The aim of this study is to compare the QoL of patients treated either with VKAs or DOACs using the Duke Anticoagulation Satisfaction Scale 25-items [17] and to evaluate if some variables as age, gender, education level, time in therapeutic range for VKAs therapy, taking medication once or twice a day for DOACs, the total daily number of medications and thrombotic and bleeding complications could have an influence on the QoL of these patients.

## 2. Materials and Methods

### 2.1. Study Design

This was a real-life prospective observational cohort study that included a total of 554 consecutive oral anticoagulated patients attending to our Hemostasis and Thrombosis Departmental Unit, which belongs to the Italian Federation of Centers for the diagnosis of thrombosis and the Surveillance of the Antithrombotic therapies (FCSA). Between January and December 2021, the DASS 25-items was administered to this cohort of outpatients as an interview. Patients on either VKAs or DOACs had an indication for long- term therapy and answered the questions after a median treatment time of 6.3 and 3.3 years, respectively. During the follow-up period, all of the patients were carefully monitored to evaluate possible bleeding and thrombotic events. 

### 2.2. Patients 

The target population included all consecutive outpatients who were on chronic therapy with VKAs or DOACs. The exclusion criteria were: age <18 years, a planned short period of oral anticoagulation or a treatment period of less than 6 months, a diagnosis of dementia or cognitive impairment and heparin therapy. 

Patients were divided into two groups: the first one included 300 consecutive outpatients on VKAs while the second comprised 254 consecutive outpatients treated with DOACs. The choice of the oral anticoagulant was made on the basis of the clinical and laboratory characteristics of each patient and the patient’s preferences after an adequate counseling. 

The patients treated with VKAs were interviewed while they were waiting for blood sampling, carried out by our nurses for the measurement of the Prothrombin Time (PT) and the medication dosage adjustment. The PT, expressed as International Normalized Ratio (INR), was performed on plasma by using an automated coagulometer (ACL TOP 550, Werfen, Barcelona, Spain) and a commercial recombinant thromboplastin (HemosIL ReadyPlasTin, Werfen, Barcelona, Spain) with an International Sensitivity Index of 1.0. 

The patients on DOACs therapy were interviewed while they were waiting for their clinical and laboratory checkup. In our Hemostasis and Thrombosis Departmental Unit a medical check was usually planned after the first, third and sixth month of therapy and then twice a year if the renal function was normal. The interval between medical examinations was reduced to three or four times a year if the patient was affected by a moderate-severe renal failure. An overview of the study and its objectives was given to all of the participants. The average time taken for each interview was 30 min and it was conducted by the same graduate student (AS), unknown to the patients, without influence their answers. In the case of patients not able to handle their oral anticoagulant therapy autonomously, the interview was administered with the help of the caregiver. The patient’s characteristics and its clinical data, recorded in a computerized medical folder, were obtained by the TAOnet^2^ software (ver. 1.2.13, EDP-Progetti, Bolzano, Italy) used in our Hemostasis and Thrombosis Departmental Unit to manage all of the oral anticoagulated patients. In particular, the variables considered were: age, gender, education level, the total daily number of medications taken, time in therapeutic range for VKAs therapy, DOACs intake one or twice a day, and thrombotic and bleeding complications. The study design was in accordance with the 1975 Declaration of Helsinki. All of the patients gave their informed consent to participate. Ethical review board approval was not requested since a periodical administration of a questionnaire is part of our clinical practice for improving patients’ adherence to therapy and for focusing the patients’ trouble in managing oral anticoagulants. Moreover, all sensitive patient data are strictly anonymous.

### 2.3. DASS 25-Items Scale 

The DASS instrument is a 25-items scale organized in three different domains that explore limitations, hassles and burdens and the psychological impact [17]. 

The first domain is made of 9 items and evaluate possible limitations about physical activity, traveling, work and medical care due to fear of bleeding, dietary restrictions and medications intake. 

The second domain includes 8 questions, from the number 10 to the number 17. It evaluates how much the oral anticoagulant therapy could affect the patient daily-life investigating hassles and burdens such as remembering to take the medicine, to wait for a blood testing or a clinical check and to manage the anticoagulant medication. 

Finally, the third domain consists of 8 items that are focused on a possible psychological impact of the oral anticoagulant therapy on patients’ life. The patients’ level of awareness of their disease is also investigated asking them whether the therapy is a reassuring factor or, on the contrary, a factor of concern. Each question has 7 possible Likert scale answers “not at all”, “a little”, “somewhat”, “moderately”, “quite a bit”, “a lot”, and “very much”. The total score obtained ranges from 25 up to 175 points. The lowest scores are associated with a better QoL, conversely, higher scores are associated with a worse QoL. Items number 17, 18, 19, 21, 23, and 25 are reverse coded before the data analysis. 

### 2.4. Statistical Analysis

The sample size calculation was done considering confidence level of 95%, a margin of error of 5% and a population proportion of 50%. The result showed that a sample size of 385 patients was required. The Medcalc software (Version 17.7.2, Ostend, Belgium) was used for data processing. Cronbach alpha was calculated for the total score and each domain to evaluate the correlation between the different items. A value of ≥0.70% was considered acceptable. 

Continuous variables were expressed as median and range since they have not a Gaussian distribution. The scores obtained by the patients’ answers to the questions of the DASS scale were expressed as geometric mean and 95% confidence intervals (95% CI) after log transformation for a clearer reading of the results. Categorical variables were expressed as frequencies and percentages. Differences between groups were assessed by the Mann-Whitney and the Fisher’s exact texts. Univariate and stepwise logistic regression analysis, expressed as odds ratios (ORs) and 95% CI, were used to calculate a possible association among the patients’ QoL and each considered clinical variable. A *p* value < 0.05 was considered statistically significant.

## 3. Results

All of the patients completed the survey and their general characteristics are reported in Table 1.

Between the two groups there were no difference as regard gender and educational level. Patients treated with DOACs were significantly older, were more frequently affected by venous thromboembolism, had a minor number of total daily medications and a shorter follow-up. 

The internal consistency of the DASS 25-items scale was good with the following Cronbach’s alpha coefficients: α = 0.90 (lower 95% CI = 0.89) for the DASS total score; α = 0.80 (lower 95% CI = 0.78) for the limitation’s domain (items 1–9); α = 0.84 (lower 95% CI = 0.82) for hassles and burdens’ domain (items 10–17) and α = 0.73 (lower 95% CI = 0.70) for the psychological impact subscale (items 18–25). 

### 3.1. Subsection

#### 3.1.1. QoL Measured by DASS Scale

In general, the QoL was better in patients treated with DOACs when compared with those on VKAs. Limitations, hassles and burdens and the daily-life psychological impact were also less important in patients on DOACs when compared with those treated with VKAs (Table 2).

As regards the limitations domain, answers to the questions 3, 4, 6, 7 and 9 showed a statistically significantly difference between patients on VKAs and those on DOACs. In fact, in patients treated with VKAs higher limitations were perceived by the patients as regard the possibility to receive medical assistance due to bleeding risk (Q3), the needs to follow a correct eating behavior (Q6) and in general about their daily-living (Q9). In patients on DOACs therapy, limitations in working for pay due to bleeding (Q4) and in following a correct drinking habit (Q7) were higher than in patients on VKAs. No differences were found as regard the other questions (Table 3).

The inconvenience and burdens were more pronounced in patients treated with VKAs when compared to those on DOACs therapy. In particular, occasional time-consuming tasks related to the monitoring of the VKAs (Q11, Q12) were the inconvenience more often declared by the patients. Moreover, the therapy was perceived as something frustrating and heavy (Q14, Q15, Q16) (Table 4).

Finally, the answers to the questions evaluating the patient’s psychological impact, showed that in those treated with VKAs, in general, the therapy had a worse psychological impact on their life (Q21, Q22), they are less satisfied (Q23) and less prone to recommend this therapy to other persons with the same medical conditions (Q25). On the contrary, patients treated with a DOACs felt worse even if they understood the reason of such a therapy (Q18) and were more scared by the risk of bleeding (Q20) (Table 5). 

#### Adverse Events

In patients treated with VKAs, 11 (3.7%) thrombotic events were recorded, of these 3 were ischemic stroke, 2 transient ischemic attacks, 3 acute myocardial infarction, 1 splenic infarction and 2 deep vein thrombosis. Hemorrhagic episodes were 31 (10%), of these 16 were gastrointestinal bleeding, 6 genitourinary bleeding, 2 post-traumatic subdural hematomas, 2 ocular hemorrhages, 1 muscular hematoma, 3 metrorrhagia and 1 hemarthrosis of the tibiotarsic joint. 

In patients treated with DOACs, 11 (4.3%) thrombotic events were recorded, of these 3 were transient ischemic attacks, 2 acute myocardial infarction, 3 ischemic stroke, 2 superficial veins thrombosis and 1 atrial appendage thrombus. Hemorrhagic episodes were 29 (11.4%), of these 15 were gastrointestinal bleeding, 8 genitourinary bleeding, 1 hemoptysis, 1 nosebleed that required nasal tamponade and 4 anemic states with a hemoglobin loss of 2 g/dL.

#### Multivariate and Stepwise Analysis Results

The results obtained from multivariate logistic regression analysis showed that, in general, in patients treated with VKAs a worse QoL was significantly associated with male sex and age >73 years. In particular daily life’s limits, hassles and burdens and the psychological impact were worse in male gender while patients older than 73 years of age only perceived great daily life’s limits. The stepwise regression analysis confirmed a statistically significant relationship among male gender and the total score of the DASS instrument (OR = 0.33, 95% CI 0.21–0.54), the limit’s (OR = 0.44, 95% CI 0.27–0.70), the hassles and burden’s (OR = 0.59, 95% CI 0.37–0.93) and the psychological impact’s domains (OR = 0.47, 95% CI 0.29–0.74).

Moreover, a relationship only existed between age >73 years and the limits’ domain (OR = 0.43, 95% CI 0.27–0.70).

As regards patients treated with a DOACs, a daily intake of more than 5 medications showed a significantly relationship with the DASS total score and the limit’s domain, highlighting a less QoL and more limitations for this reason. Moreover, a follow-up shorter than 3.3 years was associated with more daily limitations. The stepwise regression analysis confirmed a statistically significant relationship among polymedicated patients (more than 5 drugs a day) and both the total score of the DASS scale (OR = 2.05, 95% CI 1.24–3.39) and the limit’s domain (OR = 1.7, 95% CI 1.02–2.79). Moreover, follow-up shorter than 3.3 years, was confirmed as a daily-life limitation (OR = 0.55, 95% CI 0.33–0.91). Finally, thrombotic episodes and twice daily intake of DOACs therapy were retained in the stepwise model of the analysis. The data showed that not having experimented a thrombotic episode during DOACs therapy was slightly but significantly associated with more hassles and burdens (OR = 0.12, 95% CI 0.01–0.98) such as the need of a twice a day intake for apixaban and dabigatran (OR = 1.75, 95% CI 1.03–2.97).

## 4. Discussion

The DASS 25-items scale is a tool that, exploring different aspects such as limitations, hassles, burdens and the psychological impact, evaluates the QoL of patients on oral anticoagulants. Recently AlAmmari et al. have validated the DASS in an Arabic version showing a very good reliability [18]. In 2018, Stephenson et al. have reported a lower DASS score in adherent- compared with nonadherent-anticoagulated patients studied by means of the Morisky Medication Adherence Scale-8 items [19,20]. In other words, they demonstrated that higher adherence to the therapy was associated with a greater satisfaction that may positively influence the patients’ prescription-taking behavior.

In this study, the DASS instrument was used to evaluated the QoL of two groups of outpatients chronically treated with VKAs or DOACs attending our Anticoagulation Clinic. The results clearly showed that patients on long term DOACs therapy had a better QoL than patients on VKAs since the total score of the DASS instrument and the scores of each domain were significantly lower.

In VKAs patients group the limitations perceived about the risk of bleeding in getting invasive medical care should be explained by the fact that the bridging therapy [21], the change from oral anticoagulant to heparin, have to be handled by the patient. Even if in our Anticoagulation Clinic a careful counseling is done about peri-procedural management of the anticoagulant therapy, the needs to administer heparin sub-cutaneously perhaps generates fear in these patients. It is curious to note that this concern of bleeding is in line with the results of the BRIDGE study that showed that a higher incidence of major bleeding events was recorded in patients treated with the bridging therapy when compared to those who did not [22].

Obviously, this kind of limitation is not perceived by patients on DOACs therapy since the bridging therapy is not required in the pre-procedural management [10], so they do not need to manage the heparin treatment at home. 

As regards the limitations about dietary and drinking behavior perceived by both groups of patients, several studies demonstrate that patients tend to change these habits when the oral anticoagulant therapy is started. Moreover, several of them continue to perceive the VKAs intake as a dietary restriction despite adequate and careful counseling [23]. Koretsune et al. [24], using the Anti-Clot Treatment Scale, has shown a marked improvement in the perception of dietary restrictions following the transition from warfarin to apixaban. However, the limitation perceived by the patients is an interesting result since it suggests the need to renew clear explanations about dietary and drinking behavior considering that diet must be varied and free without excluding the vegetables intake and alcohol consumption should be moderate, regardless of anticoagulant therapy. It therefore seems important to establish a trustworthy doctor-patient relationship so that the pieces of information given are taken into account more than those found on the web or reported by friends and acquaintances who use the same type of medications. Only patients treated with a DOAC declared a higher limitation in working for pay due to the fear of bleeding. It is possible that not knowing their level of anticoagulation is the reason of this limitation. 

As was predictable, the main drawbacks for patients on VKAs therapy were to periodically go to the Anticoagulation Clinic to perform the blood sample for the INR checks. This kind of medical visit is considered by the patients time-consuming, heavy and frustrating. Undoubtedly, DOACs therapy has minimized the hassles and burdens of the health controls even if it has not completely avoided them. A regular follow-up is strongly advised by international guidelines especially if patients are older age, frailty and have relevant comorbidities such as renal and hepatic failure [10]. Moreover, medical checks could be a tool for lowering the variable non-adherence and persistence to DOACs intake that is reported between 38% and 99% depending on the setting considered [25]. 

At this regards, Hemostasis and Thrombosis Unit are the best dedicated landmark for this kind of patients considering the doctors’ expertise in managing oral anticoagulants and in facing thrombosis and bleeding complications. Recently, we have shown that adherence and persistence in patients treated with either VKAs or DOACs were similarly good when they were followed by a Hemostasis and Thrombosis Unit [26]. To resolve the inconveniences of patients on VKAs treatment the use of portable coagulometers can be an adequate tool. It has been demonstrated that the efficacy and safety of these point-of-care is similar to that of the conventional monitoring [27]. Several advantages are been reported such as the ease of use, since the INR test is performed on capillary blood, the speed with which the INR result is obtained and the reduction in the number of medical checks that decrease from a frequency of two to three times a month to two times a year. In our experience, the use of the portable coagulometers improves the quality of life of the patients and, as recently shown, they represent a means of ensuring the continuity of care of these patients also in emergency conditions such as the COVID-19 pandemic [28,29].

In general, the psychological impact of starting a VKAs therapy get worse the QoL and for this reason patients are less satisfied and less prone to recommend this therapy to other persons with the same medical conditions. In patients treated with a DOACs, starting such a therapy has a minor psychological impact as reported by Stephenson et al. [19]. However, in our patients, even if they understand the importance to take such a therapy, DOACs treatment triggers some concerns about the risk of bleeding perhaps since the advantage to reduce the frequency of the medical check is counterbalanced by the consciousness of unknowing their anticoagulation levels.

Male gender on VKAs treatment showed the worse QoL, more limits, hassles and burdens and a great negative psychological impact. All of these issues disappeared when males’ patients were treated with a DOACs. A possible explanation of this result is that the laboratory and clinical examinations, necessary for VKAs monitoring, were considered interfering with their work and family activities. Moreover, in almost all of the patients older than 73 years of age the limitations perceived due to VKAs could be linked to the difficulties of reaching our Hemostasis and Thrombosis Unit independently and of managing the therapy since the daily dosage could change and often the tablet should be cut. DOACs treatment were a cause of low QoL and more daily limitations only in polymedicated patients since apixaban and dabigatran require twice daily intake so contributing to increase the total daily number of medications taken. Unfortunately, our study did not have enough power to evaluate possible differences between these two subgroups of patients (DOACs intake once or twice daily) but this could be the topic of a future study.

A period of therapy shorter than three years was also associated with more daily limitations and not having had thrombotic events increased the hassles and burdens. The explanation could be that the majority of the patients were affected by atrial fibrillation and they did not ever experience an episode of ischemic stroke or systemic embolism. In their minds it can be difficult to accept to chronically take such a therapy that on one hand increases their hemorrhagic risk and, apparently, on the other does not change their clinical conditions.

This study has some limitations. The findings cannot be generalized to all of the possible settings. Oral anticoagulated patients have several options of monitoring their therapy. General practitioner, cardiologist, hematologists, internists and other specialists could take care of these patients. Moreover, self-testing and self-management is another choice to handling the therapy with VKAs. As regard DOACs treatment, often patients are not regularly followed by means of laboratory and clinical checks so lowering their adherence and persistence to the therapy. The DASS instrument could therefore give different results when applied to these various conditions. 

## 5. Conclusions

The DASS instrument is a useful tool to investigate the QoL of patients on oral anticoagulant therapy. It allows doctors at Anticoagulation Clinics to realize which are the troubles linked to this kind of therapy. VKAs appear the worse choice since they negatively influence the daily life of these patients in terms of both less satisfaction and time-consuming tasks related to the monitoring. DOACs confer a better QoL giving the advantage to reduce these time-consuming tasks but these medications seem able to induce some concerns about not knowing how their therapy is working.

Telemedicine could be an effective tool in resolving the daily-life limitations and hassles linked to both kind of therapy.

## Figures and Tables

**Table 1:** General characteristics of the patients on VKAs and DOACs therapy.

	VKAs *n* = 300	DOACs *n* = 254	*p*
Age, median (range)	72 (21–95)	77 (31–96)	<0.0001
Females, *n* (%)	139 (46%)	111 (44%)	0.54
Indications to oral anticoagulants:			
Atrial fibrillation, *n* (%)	187 (62.4%)	176 (69%)	0.09
Venous thromboembolism, *n* (%)	54 (18%)	78 (31%)	0.0006
Mechanical heart valves, *n* (%)	16 (5.3%)	-	
Antiphospholipid Syndrome, *n* (%)	31 (10.3%)	-	
Others	12 (4%)	-	
Medications:			
Warfarin, *n* (%)	100 (33.3%)	-	
Acenocoumarol, *n* (%)	199 (66.3%)	-	
Phenprocoumon, *n* (%)	1 (0.4%)	-	
Dabigatran, *n* (%)	-	37 (14.6%)	
Rivaroxaban, *n* (%)	-	69 (27.2%)	
Apixaban, *n* (%)	-	121 (47.6%)	
Edoxaban, *n* (%)	-	27 (10.6%)	
Education level:			
Low-intermediate, *n* (%)	208 (69%)	188 (74%)	0.26
High, *n* (%)	92 (31%)	66 (26%)	
Daily drugs intake:			
More than 5 drugs, *n* (%)	178 (59%)	121 (48%)	0.006
Follow-up (years), median (range)	6.3 (0.5–9.3)	3.5 (0.5–8)	<0.0001
TTR, median (range)	77% (26–100%)	-	

Legend: TTR = time in therapeutic range.

**Table 2:** Total DASS score in patients treated with VKAs and DOACs.

Drugs	Total Score	Limitations	Hassles and Burdens	Psychological Impact
VKAs	51.0, 49.2–52.8	15.4, 14.7–16.1	15.5, 14.8–16.2	18.9, 18.2–19.6
DOACs	44.7, 42.9–46.5	13.8, 13.1–14.6	12.7, 12.1–13.3	17.2, 16.5–17.9
*p*	<0.0001	<0.0001	<0.0001	0.0007

Legend: the data are presented as median, and range.

**Table 3:** Limitations in patients treated with VKAs or DOACs.

DASS Items	VKAs	DOACs	*p*
Q1 How much does the possibility of bleeding or bruising limit you from taking part in physical activities (for example, housework, gardening, dancing, sports, or anything else you would usually do)?	1.56, 1.47–1.66	1.51, 1.41–1.63	0.60
Q2 How much does the possibility of bleeding or bruising limit you from traveling?	1.26, 1.19–1.33	1.34, 1.25–1.44	0.22
Q3 How much does the possibility of bleeding or bruising limit you from getting the medical care you need (for example, visiting a dentist, chiropractor, or doctor of your choice)?	1.73, 1.61–1.87	1.48, 1.38–1.59	0.004
Q4 How much does the possibility of bleeding or bruising limit your ability to work for pay?	1.14, 1.08–1.19	1.25, 1.17–1.33	0.005
Q5 Overall, how much does the possibility of bleeding or bruising affect your daily-life?	1.64, 1.53–1.75	1.50, 1.40–1.60	0.06
Q6 How much does anti-clot treatment limit your choice of food (diet)?	1.93, 1.80–2.07	1.28, 1.21–1.36	<0.0001
Q7 How much does anti-clot treatment limit the alcoholic beverages you might wish to drink?	1.11, 1.07–1.16	1.27, 1.19–1.35	0.0006
Q8 How much does anti-clot treatment limit the over-the-counter medications (for example, aspirin, ibuprofen, vitamins) you might wish to take?	1.68, 1.56–1.81	1.68, 1.55–1.83	0.95
Q9 Overall, how much does anti-clot treatment affect your daily-life?	1.78, 1.65–1.92	1.48, 1.38–1.60	0.0008

Legend: the data are presented as median, and range.

**Table 4:** Hassles and burdens in patients on VKAs or DOACs therapy.

DASS Items	VKAs	DOACs	*p*
Q10 How much of a hassle (inconvenience) are the daily tasks of anti-clot treatment?	1.35, 1.27–1.43	1.40, 1.31–1.50	0.30
Q11 How much of a hassle (inconvenience) are the occasional tasks of anti-clot treatment?	2.85, 2.64–3.08	1.56, 1.45–1.68	<0.0001
Q12 How complicated do you find your anti-clot treatment to be?	1.39, 1.31–1.47	1.30, 1.23–1.37	0.23
Q13 How time-consuming do you find your anti-clot treatment to be?	1.74, 1.62–1.87	1.37, 1.29–1.45	<0.0001
Q14 How frustrating do you find your anti-clot treatment to be?	1.61, 1.51–1.73	1.39, 1.30–1.49	0.0017
Q15 How painful do you find your anti-clot treatment to be?	1.56, 1.45–1.67	1.42, 1.32–1.52	0.042
Q16 Overall, how much of a burden do you find your anti-clot treatment to be?	1.59, 1.49–1.70	1.40, 1.31–1.50	0.0011
Q17 Overall, how confident are you about handling your anti-clot treatment?	1.94, 1.83–2.05	1.93, 1.82–2.00	0.78

Legend: the data are presented as geometric means, and 95% CI.

**Table 5:** The psychological impact in patients on VKAs or DOACs therapy.

DASS Items	VKAs	DOACs	*p*
Q18 How well do you feel that you understand the medical reason for your anti-clot treatment?	1.87, 1.76–1.99	2.03, 1.90–2.18	0.03
Q19 How much do you feel reassured as a result of your anti-clot treatment?	2.16, 2.05–2.29	2.17, 2.05–2.29	0.94
Q20 How much do you worry about bleeding and bruising?	1.86, 1.73–2.01	2.10, 1.94–2.28	0.029
Q21 Overall, how much has anti-clot treatment had a positive impact on your life?	3.30, 3.11–3.51	2.43, 2.27–2.59	<0.0001
Q22 Overall, how much has anti-clot treatment had a negative impact on your life?	2.12, 1.99–2.27	1.79 1.68–1.92	<0.0001
Q23 Overall, how satisfied are you with your anti-clot treatment?	2.39, 2.27–2.52	2.00, 1.89–2.12	<0.0001
Q24 Compared with other treatments you have had, how difficult is your anti-clot treatment to manage?	1.33, 1.26–1.41	1.38, 1.31–1.47	0.12
Q25 How likely would you be to recommend this form of anti-clot treatment to someone else with your disease or medical condition?	2.20, 2.07–2.33	1.87, 1.77–1.99	0.0001

Legend: the data are presented as geometric means, and 95% CI.

## Data Availability

The data presented in this study are available on request from the corresponding author. The data are not publicly available due to no insertion in data repository.
